# A High‐Resolution Self‐Powered Angular Displacement Sensor Based on Triboelectric Nanogenerator With Time‐Grating

**DOI:** 10.1002/advs.76634

**Published:** 2026-07-17

**Authors:** Shuxian Wang, Donghang An, Shiyou Liu, Zhiyi Wu

**Affiliations:** ^1^ School of Integrated Circuits Chongqing University of Posts and Telecommunications Chongqing China; ^2^ School of Computer Chongqing University Chongqing China; ^3^ Chongqing Qingshan Industry Co., Ltd Chongqing China; ^4^ International Frontier Interdisciplinary Science Research Institute Beihang University Beijing China

**Keywords:** angular displacement sensors, self‐powered, time‐grating, triboelectric nanogenerators

## Abstract

In modern manufacturing and engineering, angular displacement sensors are crucial for motion control and feedback systems in Electromechanical equipment. Traditional sensors like resolvers, capacitive, and photoelectric encoders, though high in precision and resolution, face limitations in size, weight, and power supply. In this paper, we report a self‐powered angular displacement sensor (SPADS) based on the triboelectric nanogenerator principle and time‐grating technology. SPADS features a compact structure composed of a stator and a rotor. The rotor is equipped with a single row of dual‐sinusoidal copper electrodes. At the same time, the stator is made of annular fan‐shaped copper electrodes covered with a layer of PTFE material. The electrical signals generated by the friction between the stator and different rotor electrodes are modulated with high‐frequency excitation pulses to obtain signals with phase containing angular displacement information. High‐frequency time pulses serve as the measurement standard to realize the phase detection, and the angular displacement is measured by counting the time pulses. Through simulation and experimental tests, the sensor can achieve a theoretical resolution of 5.18“ and a measurement accuracy within the range of ±716”. This paper has its significance for reference to miniaturized, efficient, and reliable sensors in the field of intelligent equipment.

## Graphical abstract

A self‐powered phase‐encoding sensing paradigm enables direct motion‐to‐time information conversion, providing a compact and autonomous strategy for high resolution angular displacement measurement.

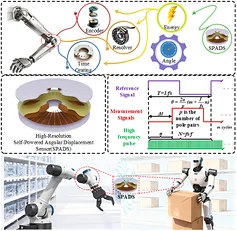

## 1 Introduction

In modern manufacturing and engineering, angular displacement sensors serve as one of the core components in motion control and feedback systems [1, 2, 3]. They consistently play a vital role in ensuring control accuracy and improving project quality across various domains such as transportation, computer numerical control (CNC) machining [4, 5], robotics, and unmanned aerial vehicles (UAVs). Particularly in the field of miniaturized intelligent equipment, including humanoid robots and UAV applications, these sensors are essential for maintaining balance and enabling stable movement.

The most widely used angular displacement sensors include resolvers [6, 7], capacitive sensors [8, 9], and photoelectric encoders [10, 11] all of which offer advantages such as high precision and high resolution. Resolver transformers operate on the principle of electromagnetic induction. The rotation of the rotor alters the coupling between the primary and secondary coils, generating standing waves whose amplitudes vary with angular displacement. After filtering out high‐frequency excitation through comparison, the angular displacement is derived using the arctangent function. Resolver transformers are known for their high reliability and strong anti‐interference capabilities. Capacitive sensors function based on the principle that the capacitance between two plates is proportional to their overlapping area. By employing a periodically patterned grid, periodic changes in capacitance are achieved during rotation, thereby determining angular displacement. These sensors are characterized by their simple structure, fast response, and good environmental adaptability. Photoelectric encoders use a beam of light passing through a coded disk with angle‐corresponding apertures. Photoelectric elements then convert this signal into a digital format to obtain the current angular displacement. They are distinguished by high precision and rapid response, with measurement accuracy and resolution primarily dependent on the light source characteristics, the number of gratings on the encoder disk, and signal processing technology. Such sensors are extensively applied in robotics and CNC machine tools. However, power supply limitations have consistently constrained efforts to reduce the machine's size and weight, as well as to extend operational endurance [12, 13]. For example, to avoid hindering the flexible movement of robots, many systems forgo external cables and instead rely on batteries to power all actuators and sensors. Yet, battery size affects both the robot's operating time and its overall weight.

Current self‐powered solutions for sensors include solar power [14, 15, 16], electromagnetic generation [17], piezoelectric generation [18, 19], and triboelectric generation [20, 21, 22, 23]. Among these, solar and piezoelectric generation exhibit instability, making them unsuitable for continuous sensor operation. Electromagnetic generation, due to its large size, is difficult to integrate with small sensors. In contrast, sensors based on the triboelectric nanogenerator (TENG) principle, introduced by Wang et al., have shown remarkable progress in applications such as energy harvesting [24, 25], displacement sensing [26, 27, 28], vibration detection [29, 30, 31], and flow monitoring [32]. Owing to their broad material adaptability, simple structure, compact size, low cost, and high integrability, TENG‐based sensors hold great potential for reducing power supply burdens, particularly in real‐time monitoring of mechanical joint displacements (Figure 1a). In recent years, various structures and materials have been proposed to enhance both the output and measurement performance of self‐powered displacement sensors. Structurally, innovations such as single‐layer grid electrodes [33], double‐layer grid electrodes [34], single‐ and multi‐layer dielectric films [35], brush‐shaped dielectrics [21], and spherical rotors [36] have been employed to improve TENG output. Material‐wise, hydrogels [37], fibers [38], and metamaterials [39] have emerged as superior alternatives to conventional TENG dielectric materials like PTFE, FEP, and PI. Recent advances in materials, composites, and device architectures have further expanded the capabilities of TENG‐based self‐powered sensors across chemical sensing, environmental monitoring, and wearable electronics applications [40, 41, 42, 43, 44, 45, 46]. These advances have led to improved voltage output, extended operational lifetimes, and added benefits such as flexibility and stretchability. Meanwhile, diverse measurement methods have been developed to enhance precision, resolution, and directional sensing. For instance, using four sets of interleaved TENG units can quadruple the resolution without compromising output intensity [47]. A structure incorporating PTFE and Kapton double‐film with double‐sided interdigitated electrodes enables simultaneous measurement of displacement and direction [34]. The combination of two such sensors allow precise two‐dimensional displacement tracking. Researchers have also modified kinematic or mechanical structures, such as using spiral springs to convert linear motion to rotation [48] or mechanical amplifiers to transform seawater buoyancy and wave energy into tensile forces driving TENG contact‐separation [24] to extract additional motion information. Nevertheless, the resolution of TENG‐based displacement sensors remains limited by electrode width and often involves a trade‐off with output intensity, which constrains the development of miniaturized high‐performance sensors. This paper proposes a measurement method that employs high‐frequency sinusoidal modulation and high‐frequency pulse injection in time‐grating technology (Note S1) to enhance the resolution of the angular displacement sensor based on TENG. A high‐resolution self‐powered angular displacement sensor (SPADS) is designed to measure the angular displacement of circular motion. This study employs dual‐sinusoidal electrodes coupled with four rectangular electrodes (take a single cycle as an example) to generate spatially orthogonal sinusoidal signals. Using a high‐frequency sinusoidal modulation method, the angular displacement is decoupled into the phase of traveling waves, thereby achieving a spatial‐to‐temporal transformation. The phase information is then measured via a high‐frequency pulse injection technique to determine the angular displacement. Through multiple experimental tests, the sensor demonstrates stable output signals while maintaining high measurement accuracy within a compact form factor. Based on calculation and experimental validation, the SPADS exhibits a maximum measurement error of no more than ±716'' and achieves a theoretical resolution of 5.18''. Unlike previous TENG‐based displacement sensors that primarily improve resolution through electrode subdivision or multi‐channel interleaving, the proposed SPADS introduces the time‐grating measurement principle into a TENG sensing architecture for the first time. The angular displacement information is transformed into the phase of a traveling wave through high‐frequency modulation, enabling spatial‐to‐temporal conversion and pulse interpolation without increasing electrode density.

**FIGURE 1:**
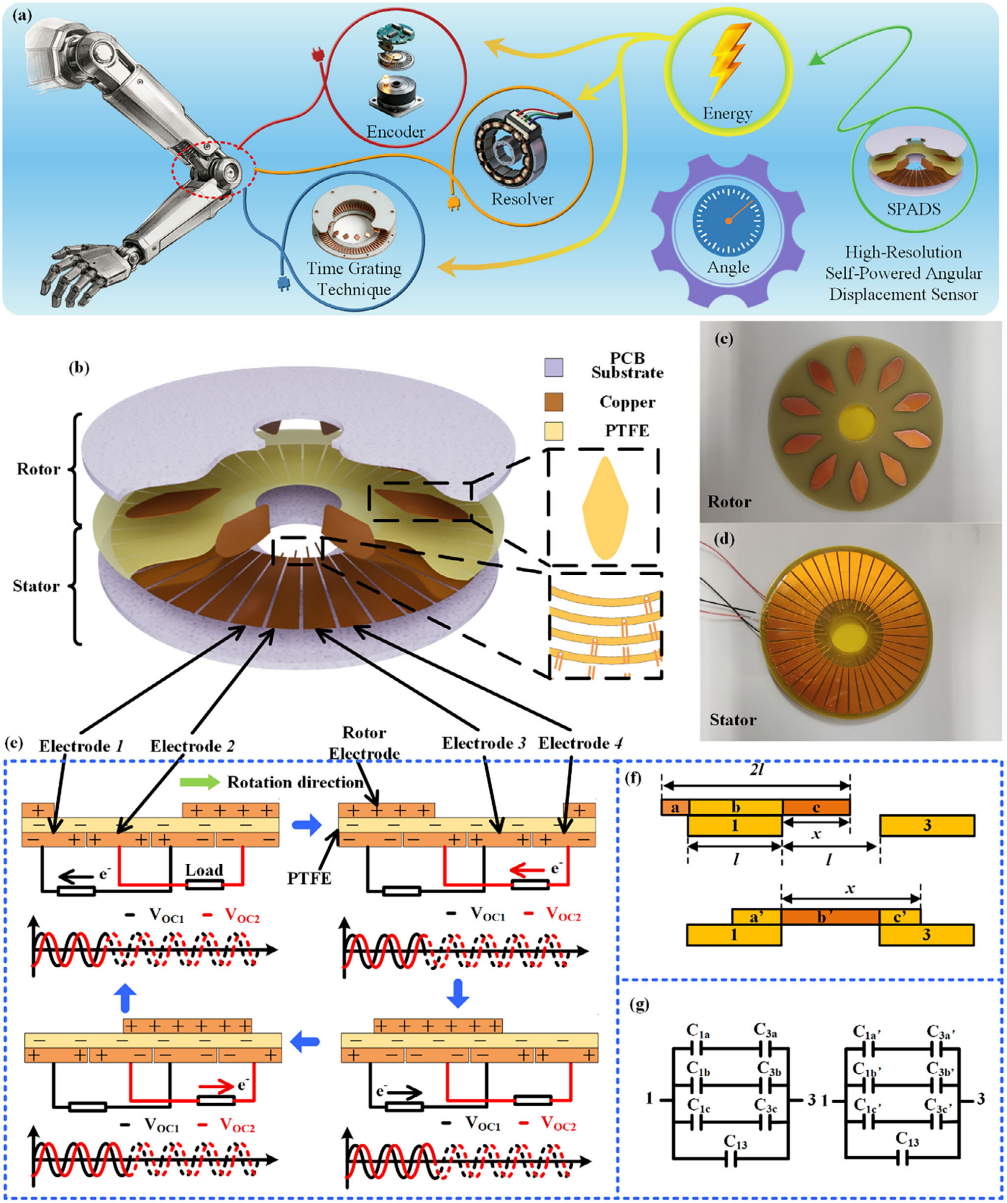
Structure and principle of SPADS. (a) Traditional angular displacement sensors require an external power supply, while SPADS can generate electricity. (b) Schematic structure of a SPADS. (c) and (d) Actual structure of SPADS. (e) Local charge behavior when the mover is at different positions during sliding. (f,g) Equivalent Capacitance of SPADS.

## 2 Results

### 2.1 Structural Configuration

The SPADS consists of a stator and a rotor (Figure 1b), both of which are manufactured using PCB technology, and there is also a layer of dielectric film covering the stator. A series of dual‐sinusoidal copper electrodes is arranged in an array on the rotor. The stator is patterned with grating copper electrodes, the number of which is four times that of the rotor electrodes. The width of each rotor electrode equals the sum of the widths of two stator electrodes and two stator electrode gaps. The electrodes on both the rotor and stator, together with the PTFE film attached to the stator, form a freestanding triboelectric‐layer‐based nanogenerator with a sliding friction structure. In the electrical connection section, every four stator electrodes are connected to form one output path, so a total of four output paths can be drawn from the stator. Two non‐adjacent electrodes are connected to an external circuit in a loop. When relative motion occurs between the rotor and stator, a pair of sinusoidal signals orthogonal to each other and correlated with the rotation angle can be obtained.

Figure 1c,d illustrates the physical structure of SPADS. Ten dual‐sinusoidal copper electrodes are patterned on the rotor, each with a width corresponding to a fan angle of 18°. Forty annular fan‐shaped copper electrodes are arranged on the stator, each with a fan angle of 8° and separated by a gap of 1°. Every four rotor electrodes are connected to four copper rings on the back as four output channels of the stator. A layer of PTFE film is applied over the stator, which not only protects the electrodes but also acts as a high‐performance triboelectric material for charge generation during relative sliding motion. In practical application, the rotor is installed on the shaft through a special coupling, and it is ensured to be parallel and coaxial with the stator fixed on the base.

### 2.2 Signal Generation Process

Figure 1e illustrates the electrical signal generation process in the SPADS. As the rotor moves, the charge on its copper electrode attracts oppositely charged particles to the upper surface of the PTFE film to achieve charge balance. Simultaneously, the stator copper electrode compensates for the charge deficit on the lower surface of the PTFE through charge flow. The potential generated in the electrode is proportional to the coupling area between the rotor and stator electrodes, and the magnitude of the current generated is related to the rotational speed of the rotor.

According to the improved capacitance model [49], the open‐circuit voltage (*V_oc_*) between the two electrodes can be calculated (Figure 1f,g). Taking the position where the right end of the rotor electrode coincides with the right end of the stator electrode 1 as the coordinate origin, and assuming that there is no gap between the stator and rotor (*d = 0*). The signal output of a pair of connected electrodes can be further simplified into two steps according to the periodicity and symmetry of circular motion: i) the rotor electrode coincides with only one stator electrode (*0<x<l*). ii) The rotor electrode coincides with both stator electrodes simultaneously (*l<x<2l*). Depending on the overlap between the electrodes, the single rotor electrodes can always be divided into three parts (*a*,*b* and *c*) with surface charges *Q_a_*, *Q_b_* and *Q_c_*, respectively, while the total capacitance between the stator electrodes consists of the capacitance between the electrodes through the surfaces *a* (*C_1a_* and *C_3a_*), *b* (*C_1b_* and *C_3b_*) and *c* (*C_1c_* and *C_3c_*) and the direct capacitance between the electrodes (*C_13_*). Therefore, the amount of charge distributed on electrode 1 can be expressed by Equation (1).

(1) $$\begin{array}{ccc} q_{1}\left(x\right) & = & q_{1a}+q_{1b}+q_{1c}\\ & = & \frac{1}{1+\frac{C_{3a}}{C_{1a}}}Q_{a}+\frac{1}{1+\frac{C_{3b}}{C_{1b}}}Q_{b}+\frac{1}{1+\frac{C_{3c}}{C_{1c}}}Q_{c} \end{array}$$

For state a), electrode *1* and the *b* part are directly opposite, and the amount of charge distributed on electrode *1* can be rewritten as Equation (2)–(5). When *d* = 0, it can be further simplified as Equation (6).

(2) $$q_{1}\left(x\right)=\frac{1}{1+Y_{a}}Q_{a}+\frac{1}{1+\frac{Z_{b}}{\sin\left(\frac{\pi x}{2l}\right)+cos\left(\frac{\pi x}{2l}\right)}}Q_{b}+\frac{1}{1+Y_{c}}Q_{c}$$

(3) $$Y_{a}=\frac{C_{3a}}{C_{1a}}$$

(4) $$Z_{b}=\;\;\frac{C_{3b}\pi d}{2w\varepsilon \sigma l}=0\left(d=0\right)$$

(5) $$Y_{c}=\frac{C_{3c}}{C_{1c}}\approx \frac{1}{Y_{a}}$$

(6) $$\begin{array}{ccc} q_{1}\left(x\right) & = & \frac{2w\sigma l}{\pi }\left[1+\frac{Y_{a}}{1+Y_{a}}\sin\left(\frac{\pi x}{2l}\right)\right.\\ & & \left.+\;\frac{1}{1+Y_{a}}cos\left(\frac{\pi x}{2l}\right)\right]\left(0<x<l\right) \end{array}$$

where *w* is the radial length of the electrode, σ is the PTFE surface charge density, *l* is the electrode width, and *ε* is the total dielectric constant. The parts that can be calculated as constants through simplification of the model can be summarized as *Z_a_*, *Z_b,_* and *Z_c_*. The ratio of the capacitance that changes due to the rotor's motion is denoted as *Y_a_*, *Y_b,_* and *Y_c_* to simplify the calculation.

In state ii), electrode *1* is opposite part *a*, and electrode *3* is opposite part *c*. Since part *b* has an equal effect on both electrodes *1* and *3*, the ratio *C_3b_*/*C_1b_* = 1. The amount of charge distributed on electrode *1* can be rewritten as Equations (7)–(10). When *d* = 0, it can be further simplified as Equation (11). Since the continuity of the charge change, *Y_a_* = 1, can be obtained, and it is finally determined that the amount of charge distributed on electrode *1* is Equation (12).

(7) $$\begin{array}{c} q_{1}\left(x\right)=\frac{1}{1+\frac{Z_{a}}{1+\cos\left(\frac{\pi x}{2l}\right)}}Q_{a}+\frac{1}{1+Y_{b}}Q_{b}\\ \;\;\;\;+\frac{1}{1+\frac{1-\sin{\left(\frac{\pi x}{2l}\right)}^{Q_{c}}}{Z_{c}}} \end{array}$$

(8) $$Z_{a}=\frac{C_{3a}\pi d}{2w\varepsilon \sigma l}=0\left(d=0\right)$$

(9) $$Y_{b}=\;\;\frac{C_{3b}}{C_{1b}}=1$$

(10) $$Z_{c}=\;\;\frac{C_{1c}\pi d}{2w\varepsilon \sigma l}=0\left(d=0\right)$$

(11) $$\begin{array}{c} q_{1}\left(x\right)=\frac{2w\sigma l}{\pi }\left[1+\frac{1}{2}\sin\left(\frac{\pi x}{2l}\right)+\frac{1}{2}\cos\left(\frac{\pi x}{2l}\right)\right]\left(l<x<2l\right) \end{array}$$

(12) $$\begin{array}{c} q_{A}\left(x\right)=\frac{2w\sigma l}{\pi }\left[1+\frac{1}{2}\sin\left(\frac{\pi x}{2l}\right)+\frac{1}{2}\cos\left(\frac{\pi x}{2l}\right)\right]\left(0<x<2l\right) \end{array}$$

Taking the charge amount at *x =* 1.5*l* as the reference charge amount, the short‐circuit transfer charge amount *Q_sc_* on electrode *1* can be calculated by Equation (13). The open‐circuit voltage between electrode *1* and electrode *3* can be calculated as the ratio of the amount of charge transferred by the short circuit to the total capacitance between electrode *1* and electrode *3*. In steps i, ii), the total capacitance is calculated from Equations (14) and (15). According to finite element simulation, *C_a_≈C_b_* can be determined, and the open‐circuit voltage between electrode *1* and electrode *3* can be obtained as Equation (16).

(13) $$Q_{sc}=q_{1}\left(x\right)-q_{1}\left(1.5l\right)=\frac{\sqrt{2}w\sigma l}{\pi }\sin\left(\frac{\pi x}{2l}+\frac{\pi }{4}\right)$$

(14) $$\left\{\begin{array}{c} \;C_{a}=\frac{1}{\frac{1}{C_{1a}}+\frac{1}{C_{3a}}}+\frac{1}{\frac{1}{C_{1b}}+\frac{1}{C_{3b}}}+\frac{1}{\frac{1}{C_{1c}}+\frac{1}{C_{3c}}}+C_{AB}\\ =\frac{C_{1a}}{2}+\frac{C_{1c}}{2}+C_{3b}+C_{13}\;\left(0<x<l\right)\\ C_{b}=\frac{1}{\frac{1}{C_{1a}}+\frac{1}{C_{3a}}}+\frac{1}{\frac{1}{C_{1b}}+\frac{1}{C_{3b}}}+\frac{1}{\frac{1}{C_{1c}}+\frac{1}{C_{3c}}}+C_{AB}\\ =C_{3a}+\frac{C_{3b}}{2}+C_{13}\;\left(l<x<2l\right) \end{array}\right.$$

(15) $$V_{oc}=\frac{Q_{sc}}{C_{total}}=\frac{\sqrt{2}w\sigma l}{\pi C_{total}}\sin\left(\frac{\pi x}{2l}+\frac{\pi }{4}\right)$$

where *C_total_* is the total capacitance between electrode *1* and electrode *3*, which can be approximated as a small constant. By shifting the coordinate origin to the left by 0.5 *L* to eliminate phase, while converting the distance *x* of linear motion into the angle *θ* of rotational motion, the open circuit voltage of SPADS is finally obtained as Equation (16).

(16) $$\left\{\begin{array}{c} V_{oc1}=\frac{\sqrt{2}\sigma wr_{m}l_{\theta }}{\pi C_{total}}\left[sin\left(\frac{\pi \theta }{2l_{\theta }}\right)\right]\\ V_{oc2}=\frac{\sqrt{2}\sigma wr_{m}l_{\theta }}{\pi C_{total}}\left[cos\left(\frac{\pi \theta }{2l_{\theta }}\right)\right] \end{array}\right.$$

where *r_m_* is the radius of the center circle of the electrode and *l_θ_* is the fan angle occupied by the Stator electrode.

The annular fan‐shaped rotor electrode allows for a range of positions with maximal overlap. In contrast, due to the dual‐sinusoidal electrode's specific geometry, the maximum overlap area occurs solely when the centerlines of the two electrodes coincide. Here, the change process of the four outputs is as follows (Demonstration with four adjacent electrodes): i) Assume a rotor electrode enters from the left end and participates in coupling. When this electrode coincides with half of electrode *1*, at this time, three‐quarters of another rotor electrode have not yet separated from coupling, and their central lines coincide with each other. In this position, electrode *2* and electrode *4* are at an equipotential, resulting in no current flow through their external load. Conversely, the potential difference between electrode *1* and electrode *3* reaches its positive maximum, driving a peak positive current in their circuit. ii) At the position of maximum coupling, where the rotor's centerline aligns with electrode *1*’s, one‐quarter of another rotor electrode remains uncoupled. At this time, the potential difference between electrode *2* and electrode *4* reaches its positive maximum, driving a peak positive current in their circuit. Conversely, electrodes *1* and *3* are at an equipotential, resulting in no current flow through their external load. iii) The rotor electrode fully enters and engages in coupling. At the position where their neutral lines coincide, the coupling area between the rotor and electrode *2* is maximized. In this configuration, electrodes *2* and *4* are equipotential, resulting in no current in their external load. Conversely, the potential difference between electrodes *1* and *3* is at its negative maximum, driving a peak negative current in their circuit. iv) When the rotor's centerline coincides with that of electrode *3*, their coupling area is maximized. Under this condition, a maximum negative potential difference between electrodes *2* and *4* drives a peak negative current in their load. Conversely, electrodes *1* and *3* are equipotential, resulting in zero current in their circuit. As a result, the SPADS can output two sinusoidal signals with a phase difference of 90°.

### 2.3 Measurement Process

Most TENG‐based angular displacement sensors convert the output signal into square wave pulses and accumulate the rotation angle by counting these pulses. Each pulse corresponds to an angular displacement equal to the width of a single electrode. Under this conversion principle, improving the sensor's resolution requires reducing the electrode width and increasing the number of electrodes. However, this approach simultaneously reduces the output voltage of the sensor. To achieve both high resolution and high output voltage, a sufficient electrode length is necessary, which increases the overall size of the sensor and is therefore incompatible with miniaturization goals.

Therefore, this paper uses a set of orthogonal high‐frequency signals to modulate the sensor output signal, and one of the high‐frequency signals as the reference signal. Then, a traveling wave signal is obtained by superimposing two orthogonal modulated signals, whose phase contains position information. Thus, a high‐frequency pulse is used to measure the phase difference between the traveling wave signal and the reference signal to obtain the angle of the rotor. The overall steps of signal processing are shown in Figure 2. First, a resistor circuit is used to make the four electrodes in the output group form a loop outside, turning them into two mutually orthogonal sinusoidal signals (Equation (17), and input them into the filter to eliminate DC bias and noise interference. The obtained sinusoidal signals pass through two separate multipliers and are multiplied with a pair of orthogonal high‐frequency signals, ultimately producing two standing wave signals (Equation (18). Then, the two standing wave signals are added to obtain a traveling wave signal (Equation (19).

(17) $$\left\{\begin{array}{c} \;u_{1}=A\;\sin\left(\omega \theta \right)=Asin\left(p\theta \right)\\ \;u_{2}=A\;\sin\left(\omega \theta +\frac{\pi }{2}\right)=A\cos\left(p\theta \right) \end{array}\right.$$

(18) $$\left\{\begin{array}{c} \;sw_{1}=A\sin\left(p\theta \right)*Bsin\left(2\pi ft\right)\\ \;sw_{2}=A\cos\left(p\theta \right)*Bcos\left(2\pi ft\right) \end{array}\right.$$

(19) $$tw=sw_{1}+sw_{2}=ABcos\left(2\pi ft-p\theta \right)$$

where *A* is the signal amplitudes, *p* is the number of output groups, *B* is the amplitude of the high‐frequency signal, and *f* is its frequency.

**FIGURE 2:**
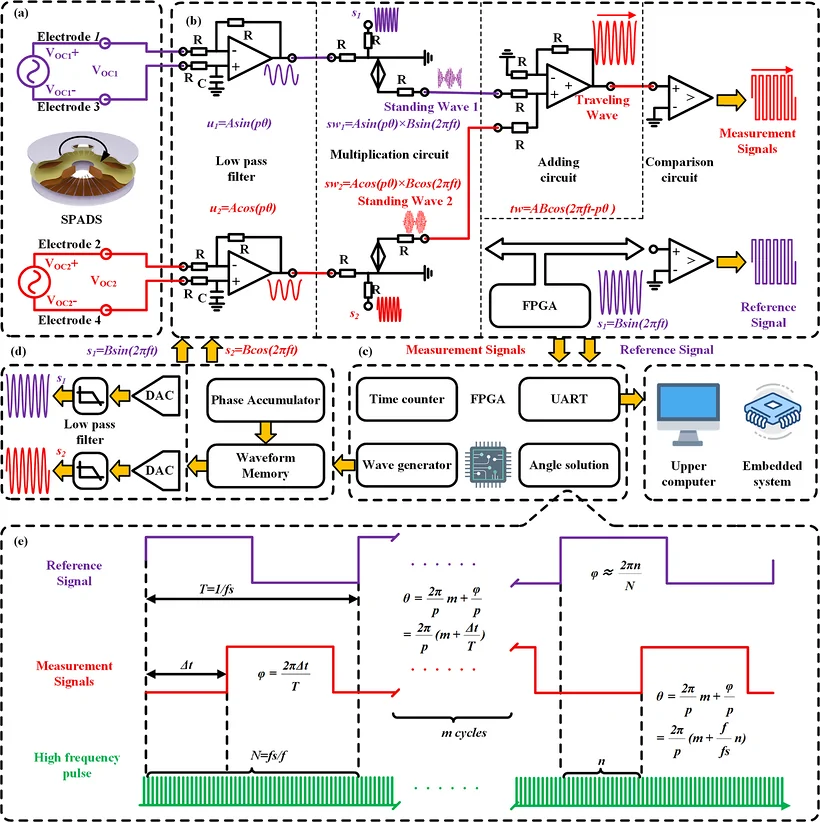
Signal processing and angular displacement calculation. (a) Quadrature sinusoidal signals generated by SPADS. (b) Signal conditioning with high‐frequency sine waves. (c) Angular displacement calculation on FPGA. (d) High Frequency Signal Generation. e) The process of angle calculation.

It is not difficult to see that the angle can be calculated by *θ* = *φ/p*, which is the ratio of the phase difference between the traveling wave signal and the high‐frequency signal and the number of output groups. In order to measure the phase difference, the traveling wave signal and the high‐frequency reference signal need to be input into the comparator, respectively, converted into square wave digital signals, and input into the FPGA. The FPGA is equipped with a 50 MHz crystal oscillator, and the time can be indirectly obtained through the number of crystal oscillator pulses. At this time, just need to start timing at the rising edge of the reference signal and end timing at the rising edge of the traveling wave signal to get the phase difference between the two signals. However, *θ* is merely an electrical angle, and its actual mechanical angle range is only 360°/p. Therefore, the counter m is used to record the number of electric angles rotated, in order to ensure that the measurement range can cover an entire rotation period. The adjusted method for calculating angular displacement is Equation (20).

(20) $$\theta =\frac{2\pi }{p}\;m+\frac{\varphi }{p}=\frac{2\pi }{p}\;\left(m+\frac{f}{f_{s}}n\right)$$

 Where *f_s_* is the crystal frequency or counting frequency, *m* is the number of counts in electrical angle, and *n is* the number of clock counts used to measure the phase difference. The theoretical resolution of the sensor is finally calculated by *4πf/pf_s_*. Therefore, when the stator structure has 10 pairs of poles and the modulation frequency is 5 kHz, the theoretical resolution of SPADS reported in this article is 5.18''. However, in practical applications, the measurement resolution is also affected by the bit width of the calculation and the accuracy of the comparator circuit. As a result, the actual value is higher than the theoretical value.

### 2.4 Optimization

In order to verify the theoretical model, we simulated the shape of this signal using COMSOL finite element simulation software(Figure 3b and the model used for simulation is Figure S1). A two‐dimensional model is used to illustrate the coupling relationship between charges during the motion process in Figure 3a.

**FIGURE 3:**
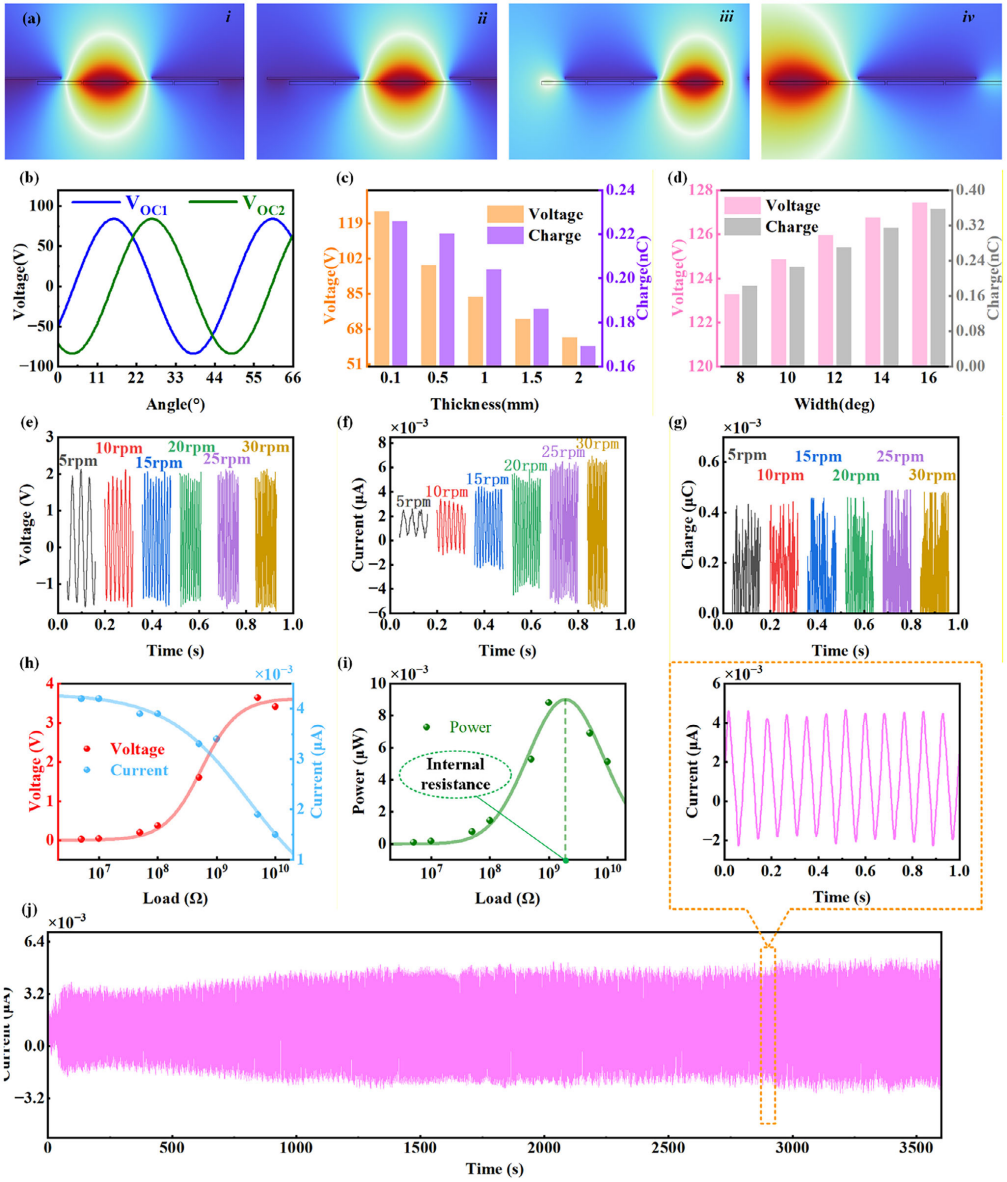
Output performance. (a) Potential simulation when the mover is at different positions during sliding. (b) Output curve using dual‐sinusoidal rotor electrode. (c) Open‐circuit voltage and transfer charge of SPADS with different dielectric film thicknesses. (d) Open‐circuit voltage and transfer charge of SPADS with different electrode widths. (e–g) Output characteristics of SPADS at different speeds. (h,i) Resistance dependency of the output current, voltage, and power of the SPADS. (j) The output stability of the short‐circuit current of SPADS at 10 rpm.

The output signal of the SPADS is influenced by the thickness of the PTFE film and the electrode area. To investigate their specific effects on signal output, we conducted numerical simulations to evaluate the voltage output under variations in PTFE film thickness and electrode width. The simulation results are presented in Figure 3c and Figures S2 and S3. As the thickness of the PTFE film increases from 0.1 to 2 mm, the output potential difference exhibits a monotonic decrease. This behavior occurs because the maximum charge carried on the rotor electrode surface is fixed, leading to a specific amount of opposite charge accumulating on the upper surface of the PTFE due to the influence of the rotor's charge. Increasing the film thickness raises the total amount of movable charge within the material, which in turn reduces the amount of charge transferred from the lower surface. As a result, the potential induced in the stator copper electrode decreases. Therefore, to maximize signal output, the PTFE film should be made as thin as possible. Regarding electrode width, simulations were performed for five values: 8°, 10°, 12°, 16°, and 18°. As shown in Figure 3d and Figures S4 and S5, the output voltage increases monotonically with electrode width, which is consistent with theoretical expectations. However, excessively large electrode widths are not desirable, as they reduce signal resolution. Alternatively, similar output performance can be achieved by increasing the electrode length while maintaining resolution.

### 2.5 Output Performance

To characterize the output performance of the SPADS under different rotational speeds and load conditions, we conducted laboratory tests using a servo motor and an electrometer. The output voltage, short‐circuit current, and transferred charge of the SPADS under different rotational speeds are shown in Figure 3e–g. Speed has little effect on open‐circuit voltage, which is only determined by the coupling area between the stator electrode and the rotor electrode. The total amount of charge transferred is determined by the electrode, the material, and the thickness of the dielectric film. As the rotational speed increases, the coupling frequency between the stator and rotor electrodes rises, accelerating charge transfer in the external circuit and thereby increasing the current. The peak current increases with the rotational speed, reaching values of 0.0042, 0.0089, and 0.0119 µA at 10, 20, and 30 rpm, respectively. It should be noted that the measured result is influenced by the sampling rate of the data acquisition system. The recorded peak tends to be slightly lower than the actual value, and this discrepancy becomes more pronounced at higher speeds, eventually leading to a decline in the measured peak when beyond a certain speed threshold. Evaluate the on‐load capability of the SPADS, various impedances within the range of 1 MΩ to 10 GΩ were incorporated into the test circuit. Tests conducted at 10 rpm revealed that within this load range, the peak output voltage follows an *S*‐shaped increasing trend, while the peak current shows an *S*‐shaped decreasing trend(Figure 3h and Figure S6). At an external load of 5 GΩ, the peak voltage reached 3.6376 V, and the peak current was 0.0019 µA. Meanwhile, the output power of the SPADS increases first and then decreases with increasing external load, peaking at approximately 2 GΩ with a value of 0.009 µW (Figure 3i). To assess the long‐term stability of the SPADS, a 3600‐second stability test was performed, as illustrated in Figure 3j. The test results show that the output voltage remained virtually unchanged throughout the 3600s, demonstrating its outstanding output stability.

### 2.6 Measurement Performance

To demonstrate the measurement performance of the SPADS in angular displacement, we employed a programmable servo motor to drive the rotor of the SPADS in a predefined motion sequence and used LabVIEW for signal processing and computation. The motion protocol was set as follows: i) rotate 60° in the reverse direction at 60 rpm and pause for 1 s; ii) rotate 90° in the reverse direction at 60 rpm, pause for 1 s, and repeat this cycle three times; iii) rotate 100° in the positive direction at 120 rpm, pause for 1 s, then rotate 260° in the positive direction at 180 rpm, and finally return to the initial position. Throughout the test, the SPADS accurately responded to various states, including different speeds, rotation directions, starts, and stops. As shown in Figure 4b and Figure S7, the detection results from the SPADS exhibit strong consistency with the motor's motion profile. The linear relationship between output frequency and rotational speed is illustrated in Figure 4c. The frequency exhibits excellent linearity with rotational speed (Correlation coefficient R^2^ = 0.9998), indicating that the signal frequency of the SPADS correlates linearly with the external trigger frequency and is suitable for subsequent angle measurement.

**FIGURE 4:**
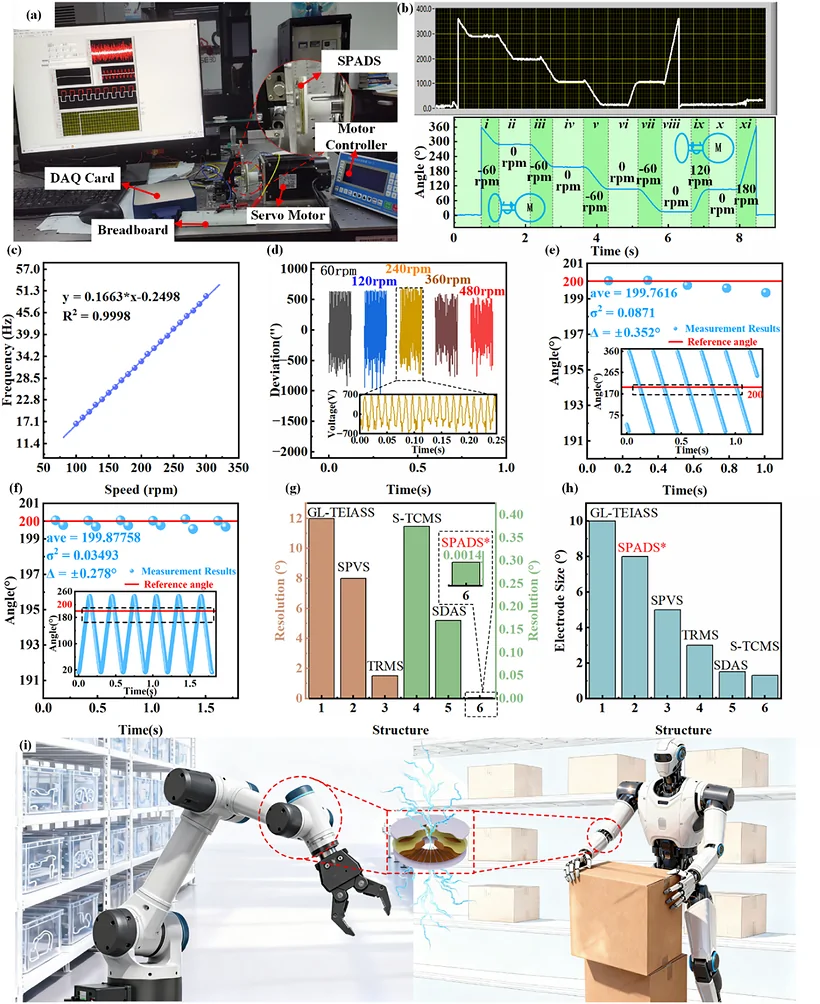
Measurement performance. (a) Photograph of the test system. (b) Displacement measurement effect of SPADS. (c) Matching the degree of output frequency and rotation speed. (d) Displacement deviation measured at different speeds. (e) Repeated measurement error during constant motion. (f) Repeated measurement error during reciprocal motion. (g,h) Comparison of resolution and electrode size among similar sensors. (i) Application of SPADS.

To further verify the measurement accuracy, tests were conducted at different rotational speeds with signal processing circuits (Figure S8) to obtain measurement data over a full rotation (Figure 4d). As noise shifts toward higher‐frequency bands, the measurement error of the SPADS decreases slightly with increasing rotational speed but generally remains around ±700″. At 240 rpm, excluding occasional larger deviations caused by system vibration, external noise, and other disturbances, the maximum error was ±716″. Additionally, through uniform rotation at 300 rpm (Figure 4e) and reciprocal motion tests (Figure 4f), the repetitive positioning error under uniform motion was measured as ±0.352° relative to a 200° reference, while the repetitive positioning accuracy under reciprocal motion was ±0.278°. The theoretical resolution is determined by the phase measurement capability of the FPGA‐based time‐grating system, fundamentally limited by the 50 MHz crystal oscillator frequency and the number of output groups. In contrast, the experimental measurement error reflects the overall accuracy under real operating conditions, influenced by mechanical misalignment, non‐uniform PTFE film thickness, electrode manufacturing tolerances, signal noise, and system vibrations. The error distribution over a full 360° rotation (Figure 4d) exhibits a periodic pattern correlated with the cyclic electrode arrangement. The error magnitude varies with angular position but remains within ±716″ across the entire range, confirming consistent measurement capability without systematic error accumulation.

For further performance context, conventional optical encoders typically achieve resolutions ranging from sub‐arcsecond to several arcseconds and arcsecond‐level accuracy but rely on external power supplies with substantial power consumption and occupy moderate to large diameters. Brushless resolvers offer accuracies in the range of tens to hundreds of arcseconds with superior mechanical robustness yet demand AC excitation power and generally exhibit higher power draw and larger physical volume than optical encoders. In comparison, the SPADS achieves a theoretical resolution in the arcsecond range without any external power supply, operates within a compact diameter, and generates its own sensing signal through triboelectric coupling. While its current experimental accuracy remains on the order of hundreds of arcseconds, the self‐powered operation and small form factor provide distinct advantages for applications where power availability and space are severely constrained, such as in untethered robotics and UAVs.

In comparison with other displacement sensors based on TENG, SPADS demonstrated its high‐resolution measurement performance in a small volume. (Table 1 and Figure 4g,h). In order to facilitate the comparison of different sensors more easily, we refer to the ratio between the resolution and the electrode width as the resolution conversion rate, which is used to measure the relationship between the measurement resolution and the sensor size. A smaller value indicates that the sensor can achieve higher resolution in a smaller volume. The results show that SPADS has great potential for application in industrial robotic arms and robots (Figure 4i).

**TABLE 1:** Performance of SPADS among similar sensors.

Reference	Resolution	Deviation	Electrode size	overall size	Resolution conversion rate
TRMS [50]	1.5°	<0.8%	sector angle 3° length N/A	N/A	0.5
S‐TCMS [47]	0.375°	<0.42°	1.3°×10mm	Inner diameters 150 mm outer diameters 190mm	0.29
dsTENG [34]	250µm	<1%	500µm × 2.5cm	20cm×6cm×1.5mm	0.5
SDAS [51]	0.17°	N/A	sector angle 1.5° length N/A	60mm×37.8mm×7.5mm	0.11
GL‐TEIASS [52]	12°	<0.731%	sector angle 10° length N/A	N/A	1.2
SPVS [53]	8°	N/A	sector angle 5° length N/A	Diameters 6.2 cm	1.6
Landslide Displacement Sensor [54]	3mm	< 3%	3 mm × 15 mm	N/A	1
US‐TDS [55]	0.0382mm	<1%	1.25mm	155 mm × 100 mm × 94 mm	0.03
SPADS*	5.18''	<716''	8°×16mm	Diameters 50mm	1.8e‐4

## 3 Conclusion

In this study, we report a high‐resolution self‐powered angular displacement sensor (SPADS) that integrates the freestanding triboelectric nanogenerator (TENG) operating in the independent layer mode with the time‐grating measurement principle. By transforming coupled sinusoidal signals into a phase‐modulated traveling wave, the proposed SPADS overcomes the long‐standing trade‐off between resolution and output voltage inherent in TENG‐based displacement sensors. The correlation between the geometry of rotor electrodes and the resulting output signal waveform was systematically investigated, with explicit analytical formulations derived. Through structural design and optimization, the SPADS employs a dual‐sinusoidal rotor electrode configuration paired with interleaved fan‐shaped stator electrodes, generating orthogonal sinusoidal signals at a coupling ratio of 1:4. Experimental results demonstrate that the sensor achieves a resolution of 5.18″ and a maximum measurement error of ±716″, surpassing the performance of existing TENG‐based displacement sensors. These findings indicate the significant potential of TENG‐based sensing technology in industrial applications, while its self‐powered nature offers novel possibilities for robotics, unmanned aerial vehicles, and related fields.

## 4 Methods

### 4.1 Fabrication of the TENG

The base of SPADS has an outer diameter of 60 mm and an inner diameter of 13 mm. On the stator, the annular fan‐shaped electrode was designed with inner and outer radii of 12 and 28 mm, corresponding to a radial width of 16 mm. The electrode has a sector fan angle of 8° and is separated from the other electrode by a 1° gap. Each electrode is sequentially connected to four copper rings on the backside of the substrate through via holes. These concentric rings, with radii of 8, 9, 10, and 11 mm, each have a width of 10 mil. When drawing rotor electrodes, it is necessary to use Excel and CAD to convert the parameterized point matrix into a polygon. The rotor electrodes are dual‐sinusoidal in shape, with an amplitude of 7 mm, and occupy a sector angle of 18° on the circumference.

There are a total of 10 such electrodes evenly distributed on the rotor substrate. Therefore, the outer and inner contours of the electrode are defined by the parametric equations *x* = cos(θ*180/*π*)*(±7*sin(10*θ*180/*π*)+20) and *y* = sin(θ*180/*π*)*(±7*sin(10*θ*180/*π*)+20), respectively. Here, *θ* is used in degrees.

### 4.2 Characterization and Measurement

The output voltage and current were measured with a Keithley 6514 electrometer. The output signals were acquired by an NI PCI‐6259 data acquisition card and processed using the LabVIEW software platform. An AC servo motor(80ST‐M02430LB) is used to drive the movement of the rotor. The positional relationship between the stator and rotor is adjusted by a small three‐axis slide.

## Author Contributions

**Shiyou Liu**: investigation, data curation, formal analysis. **Zhiyi Wu**: conceptualization, methodology, writing – review and editing, formal analysis. **Shuxian Wang**: conceptualization, methodology, funding acquisition, writing – original draft. **Donghang An**: data curation, writing – original draft, software.

## Funding

This research was funded in part by the Natural Science Foundation of China under Grant 52205553, in Part by the Science and Technology research Project of Chongqing Education Commission (Grant No. KJQN202300628 and KJQN202500641), and in Part by the Chongqing University of Posts and Telecommunications research Fund (Grant E012A2021210).

## Conflicts of Interest

The authors declare no conflicts of interest.

## Data Availability

The data that support the findings of this study are available from the corresponding author upon reasonable request.
